# A Practitioner in Derbyshire, in Answer to Crito

**Published:** 1807-11

**Authors:** 


### *To the Editors of the Medical and Physical Journal.*

Gentlemen,

I Am charged by Crito of perverting his and Dr. Cayley's meaning, in my Remarks on their respective Papers; a charge, which, with your permission, I cannot silently pass over. I may not, perhaps, have adhered to the very words of Crito's paper, but, on a careful review, I cannot think that I have perverted its sense. The challenged passage is, where I have observed, that Crito attributes every thing bad to compression. "Where," says he, "does he find me to say that?" Not, I will allow him, in the exact terms; but, does he not in his case of wounded ulnar artery, represent the arm as swelled equal to a man's thigh, the wound looking ill with a gangrenous appearance? And does he not immediately say, that he inserted this case to show the effects of compressing the artery? And further, "the swelling was very alarming of itself without the hæmorrhage," which he could only attribute to compression. There is therefore, it will appear, a difference only in words between us, not amounting, I hope, to a perversion of sense.

I will not proceed further with the dispute between us, respecting the propriety of calling the tibial arteries branches merely of the popliteal. I only meant to inculcate cate the importance of these arteries;—while Crito observes, " Allowing it to have been aneurism, it was only one of the branches of the popliteal." Evidently derogating from the importance of these arteries. But what vessels could he select of more consequence to the practical surgeon, considering their liability to accidents, and, in some points of them, their difficulty almost amounting to impossibility of access? We have already observed on this head, that it would have added greatly to the interest of Dr. Cayley's case, if he had given us the precise situation of the wound; we could then have spoken more determinately about, not only that, for it would besides have been very satisfactory to have known what part of the artery it was, that Dr. Cayley was so fortunate as to obliterate by compression. It being a fact, of which we all must be apprised, that those points of the arteries that are in a manner beyond the reach of operation, would, from being so surrounded and cushioned as it were with flesh, be the most difficult to obliterate by compression. Crito indeed, still holds an opinion, that this was not a wound of the anterior tibial artery; a point, very unnecessary for us to establish now, or we would refer him, in support of it, to the consideration of the first gush of blood to the nature of the instrument which gave the wound, and would ask of him, what other artery have we in the track of this, that could afford us a tumour of considerable size, in which, " the pulsation through the whole was not only tangible, but visible across the room?" Indeed, " of this circumstance," as Crito observes by me, " Dr. Cayley must have been the best judge."

Crito now recurs to his case of wounded ulna artery, and laments, that we should not understand what he means by sewing the artery; and to be sure, he has given us a luminous explanation. The case runs thus, " On the second day it began again to bleed. He (the patient) then came into town, and had the artery sewed by a surgeon. On the fourth day after it was sewed, the artery began to bleed; an attempt was again made to secure it, with very little hope of success; it, however, prevented the recurrence of the hæmorrhage for three days, but on the fourth day it again returned; any attempt to sew it was now deemed improper."—Would any body suppose, in all this mention of sewing, that Crito was diving, time after time, with the crooked needle, to hook, as it were, the perverse creature, and include it in a ligature? And yet his explanation brings us to such conclusion. That he did not however, in in his respeated attempts, get round the artery, is self evident, from the frequent and quick returns of hæmorrhage, as well as from the man requiring at a future time, a certificate to exempt him from military duty, which Crito, observing his perfect recovery, denied him. Whereas, if he had succeeded in getting round the artery in this way, that is, by determined stroke of the needle, he must unavoidably have encompassed the nerve, and in that case, the contracted fingers of the sufferer would have exhibited a sad and unequivocal testimony of his inability.

Finally, another case of this sort is alluded to, "Where the ulnar artery was cut with a knife, which terminated fatally." The case, we are told, was under the direction of men " of professional consequence."—Crito, with an appearance of pleasantry, now goes on to observe, " Had the Derbyshire Practitioner been called, he would have obliterated the artery by a single pull." What the Derbyshire Practitioner might have done, he cannot say, but though himself of no professional consequence, he can assure Crito, that it would be a matter of painful consideration with him, to have a case of wounded ulnar artery simply, terminate fatally.

A PRACTITIONER IN DERBYSHIRE.

*October*, 11, 1807.

